# Diagnosis and management of breast implant capsule recurrence following mastectomy and subpectoral implant – innovative use of ADM for reconstruction

**DOI:** 10.1093/jscr/rjac432

**Published:** 2022-10-10

**Authors:** Chien Lin Soh, Christian M Asher, Parto Forouhi, Penelope Moyle, Nuala Ann Healy, Charles M Malata

**Affiliations:** University of Cambridge School of Clinical Medicine, Addenbrooke’s Hospital, Cambridge, UK; Department of Plastic and Reconstructive Surgery, Cambridge University Hospitals NHS Foundation Trust, Addenbrooke's Hospital, Cambridge, UK; Cambridge Breast Unit, Cambridge University Hospitals NHS Foundation Trust, Addenbrooke's Hospital, Cambridge, UK; Cambridge Breast Unit, Cambridge University Hospitals NHS Foundation Trust, Addenbrooke's Hospital, Cambridge, UK; Cambridge Breast Unit, Cambridge University Hospitals NHS Foundation Trust, Addenbrooke's Hospital, Cambridge, UK; Department of Plastic and Reconstructive Surgery, Cambridge University Hospitals NHS Foundation Trust, Addenbrooke's Hospital, Cambridge, UK; Cambridge Breast Unit, Cambridge University Hospitals NHS Foundation Trust, Addenbrooke's Hospital, Cambridge, UK; Anglia Ruskin University School of Medicine, Cambridge, UK

## Abstract

It is well reported that patients who have undergone breast augmentation and subsequently develop breast cancer can successfully undergo breast-conserving therapy with preservation of their implants. However, there is a paucity of literature on the radiological investigations and surgical techniques in postmastectomy implant-reconstructed patients who develop recurrences to enable preservation of their implant-based reconstruction whilst effectively treating the local recurrence. The wide adoption of acellular dermal matrix use in prosthetic breast reconstruction in recent years has made radiological evaluation of such patients challenging. Herein presented is a case of a 37-year-old woman where wide local excision of a local recurrence abutting a peri-implant capsule following previous mastectomy and implant-acellular dermal matrix (ADM) reconstruction was performed with successful preservation of reconstruction volume (and shape) using an ADM patch to repair the capsular defect whilst retaining the implant *in situ.* Radiological investigation facilitated and guided the surgical planning and oncological clearance.

## INTRODUCTION

Local cancer recurrence in the context of a previous breast reconstruction can pose a challenge in diagnosis and management [1]. There is a paucity of literature on the radiological investigations and surgical techniques in postmastectomy implant-reconstructed patients who develop recurrences to enable preservation whilst effectively treating the local recurrence.

## CASE REPORT

**Figure 1:**
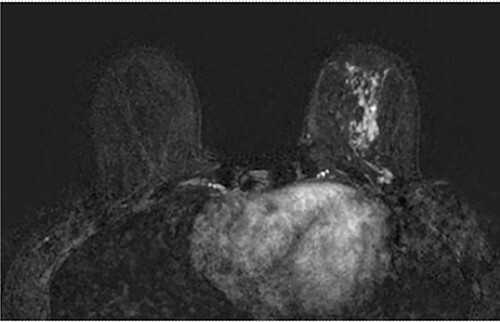
Axial fat-saturated post contrast Magnetic Resonance Imaging (MRI) of the breasts. There is extensive stippled non mass like enhancement throughout the inferior and central left breast with type 1 benign kinetics in keeping with DCIS.

A 37-year-old Caucasian woman underwent a left therapeutic skin-sparing mastectomy and sentinel lymph node biopsy and a simultaneous right risk-reducing skin-sparing mastectomy in 2016. She had bilateral immediate subpectoral breast reconstruction with Becker-35 expandable implants combined with SurgiMend® (Integra LifeSciences, Princeton, NJ) acellular dermal matrix (ADM). Histology revealed 45 mm of high-grade ductal carcinoma *in situ* (DCIS) with three foci of invasive carcinoma No Special Type (NST) measuring 4, 2 and 1.6 mm in size (Fig. 1). The disease was ER positive, PR-negative and HER-2 negative, the two sentinel nodes were disease-free and the patient received tamoxifen as adjuvant therapy.

**Figure 2:**
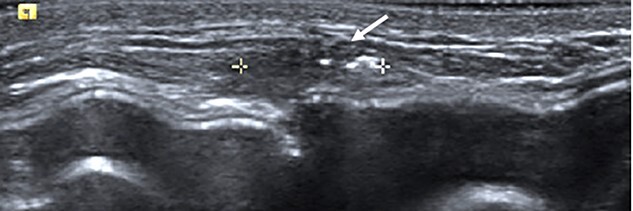
Ultrasound of the left central breast identified an irregular mass on the capsule of the implant with a focus of hyperechoic calcification (arrow).

**Figure 3:**
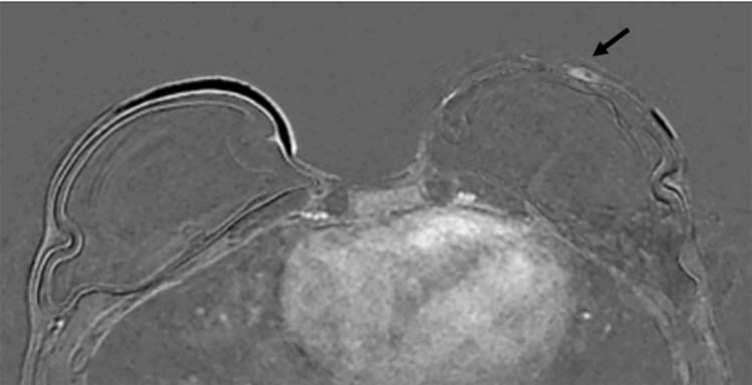
Axial post contrast fat-saturated sequence identified bilateral implants and an 11 mm oval foci of type 1 enhancement in the site of clinical concern on the capsule (black arrow).

Clinical findings during follow-up were unremarkable until 2020, when she represented with a new lump in the reconstructed left breast neighbouring the mastectomy scar inferiorly. A 1 cm subcutaneous lump with no skin tethering, was palpable along the uniting suture line of the ADM-pectoral muscle. Imaging with ultrasound was indeterminate. This implant proximity rendered it unsuitable for core biopsy due to the high risk of iatrogenic implant perforation (Fig. 2). Subsequent MRI (Fig. 3) illustrated an 11 mm low signal lesion correlating clinically to the palpable lump with mild enhancement but indeterminate appearance.

A diagnostic narrow margin excision biopsy was performed under local anaesthesia with histological assessment confirming invasive high-grade DCIS extending to multiple margins, ER positive and HER2 negative, mirroring the histology of the primary tumour. MDT recommendation was for therapeutic wide excision, adjuvant radiotherapy and axillary staging. An ultrasound scan of the left axilla demonstrated a single node with an enlarged eccentric cortex, core biopsy of this node showed no evidence of malignancy. She underwent wide local excision of the recurrence with a 1 cm margin around the diagnostic biopsy scar, excising a composite of skin, scar and the full thickness of underlying implant capsule under direct vision, without injury.

The resultant composite defect was reconstructed with an 8 cm × 8 cm piece of SurgiMend® ADM (Integra LifeSciences, Princeton, NJ) anterior to the implant, with edges deep to the capsule (similar to an inlay mesh hernia repair) to ensure full coverage of the exposed implant and reinforce its coverage. The ADM was secured with 2/0 interrupted monofilament absorbable polydioxanone (PDS) sutures (*Ethicon*, Edinburgh, UK). Final histology confirmed complete clearance at all margins and the patient underwent adjuvant chest wall radiotherapy with 26 Gys over a week in five fractions, with subsequent maintenance on with Tamoxifen for 5 years.

**Figure 4:**
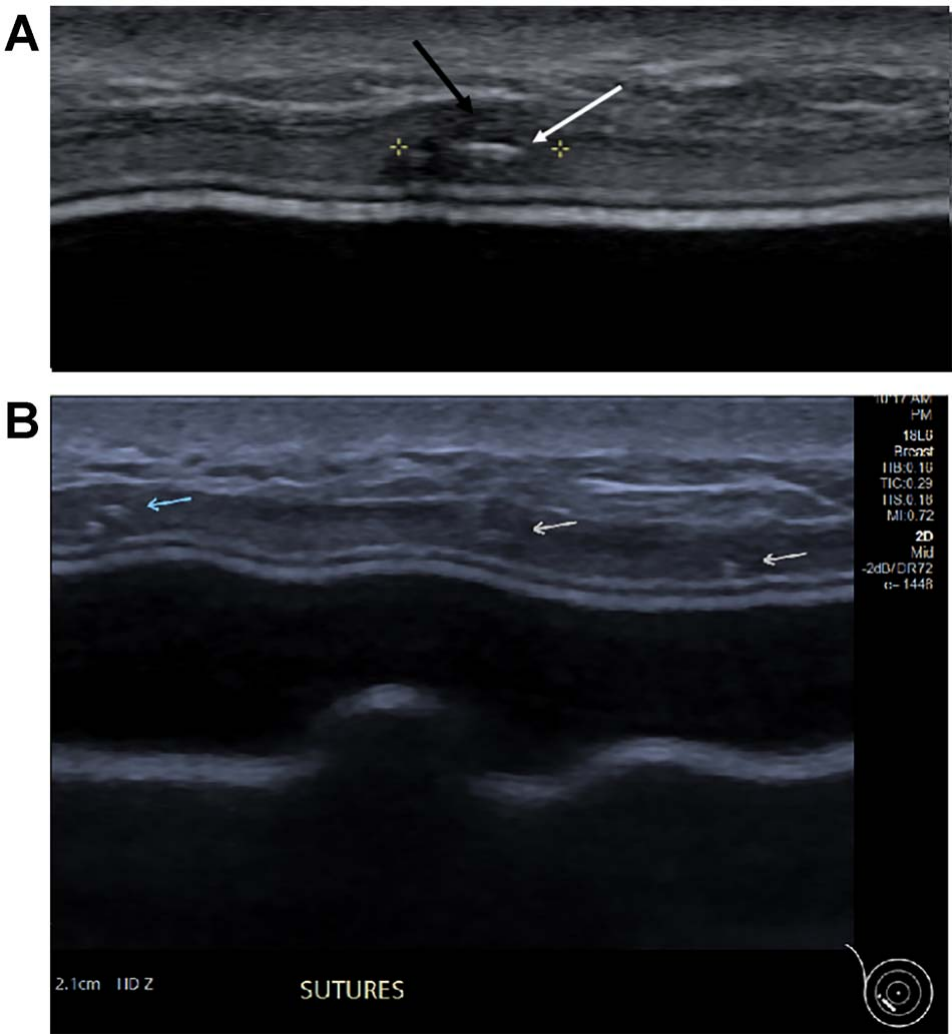
(**A**) Magnified ultrasound image identified a 5 mm hypoechoic focus of thickening within the capsule (black arrow and area between the cursers) with two parallel lines centrally within the focus which represents the suture (white arrow). (**B**). The ultrasound demonstrates the equally placed sutures identified as small white parallel lines (arrows) across the ADM.

Three months postoperatively, the patient presented to the breast clinic with a nodule on the lower part of her left reconstructed breast deep to her (wide excision) scar. Ultrasound revealed an indeterminate nodule with a bright echoic centre. Differentials included the unabsorbed knot of an internal suture or possible local recurrence. Dynamic contrast breast MRI, however, did not demonstrate any abnormal enhancement. Ultrasound undertaken along with the operating surgeon demonstrated the patch of ADM inserted at the time of initial surgery as a thickening, with three identical regularly placed nodules measuring 3–5 mm corresponding to PDS sutures (Fig. 4), thus avoiding unnecessary surgery. Four months post-operatively, the nodule was no longer palpable.

**Figure 5:**
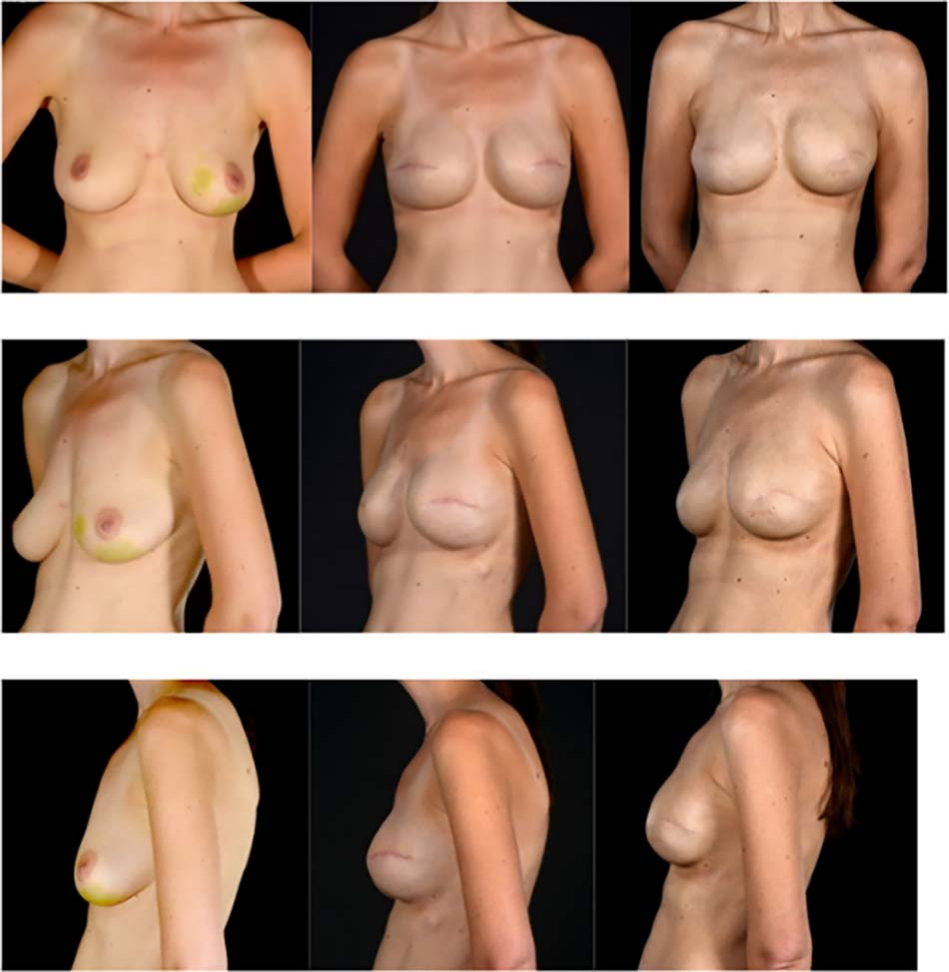
Pre and post photographs showing the preservation of reconstructed breast volume and shape despite the wide local excision of a recurrence and the peri-implant capsule shape despite the wide local excision of a recurrence and the peri-implant capsule. (**A**) (first column of images) images are pre-mastectomy, (**B**) column images are 4 years later prior to WLE and (**C**) column images are after WLE of the recurrence and reconstruction of the capsule deficit with a surgimend ADM patch. Note the maintenance of the shape and size of the breast.

Six months following adjuvant radiotherapy, the patient had retained size and shape of the reconstructed breast but predictably showed early signs of capsular contracture (Baker grade 2) (Fig. 5).

## DISCUSSION

Local cancer recurrence in the context of a previous breast reconstruction can pose a challenge in diagnosis and management [1]. There is concern that the presence of a reconstruction may mask local cancer recurrence [2]. There is little consensus on the frequency of routine radiological surveillance of these patients as well as on the surgical treatment of these recurrences [3, 4]. However, many patients who develop breast with implants *in situ* can undergo breast-conserving surgery with implant preservation [5, 6]. This case report addresses previous cancer patients reconstructed with breast implants who then develop tumour recurrence overlying the implant and radiological investigations and surgical techniques in breast conservation when a patient already has implants. An institutional review of 52 patients with prior cosmetic breast implants undergoing breast conservation surgery for breast cancer demonstrated equivalent success in excision versus those with no implants [7].

This report illustrates a technique for reconstruction following wide local excision of a local recurrence including the peri-implant capsule facilitating preservation of the implant *in situ* and maintaining the reconstruction shape and volume by repairing the capsule defect with the use of ADM, and it helped to maintain implant pocket volume and promote a good aesthetic outcome for the patient [8, 9].

This case report also highlights the challenges in the diagnosis of lesions that are closely associated with implants. MRI interpretation can be challenging when differentiating between local recurrence and postoperative changes such as granulomas associated with sutures as both can have enhancement including type 1 kinetics [10, 11]. This can be a particular problem when non-absorbable or long-lasting sutures are used, which is demonstrated in a previous case report describing sutures masquerading as a breast lesion [12]. MRI is beneficial in identifying the extent of disease for surgical planning.

Ultrasound can demonstrate excellent views of the implant [13]. It is important for the radiologist to fully understand the surgical techniques involved in cases of breast reconstruction and to bear in mind suture position when reporting the images. It is incumbent on surgeons to give the radiologists adequate information as to the previous surgical procedures and highlight any differential diagnoses to them in the radiology request.

## CONCLUSION

In summary, we present a case of wide local excision of a local recurrence in an implant capsule following mastectomy and implant-reconstruction with successful subsequent preservation of reconstruction volume with an ADM ‘mesh’ patch.

## References

[1] Mirzabeigi MN , Rhemtulla IA, McDonald ES, Sataloff DM, Kovach SJ, Wu LC, et al. Locoregional cancer recurrence after breast reconstruction: detection, management, and secondary reconstructive strategies. Plast Reconstr Surg 2019;143:1322–30. 30789475 10.1097/PRS.0000000000005522

[2] Kropf N , McCarthy CM, Disa JJ. Breast cancer local recurrence after breast reconstruction. Handchir Mikrochir Plast Chir 2008;40:219–24. 18716989 10.1055/s-2008-1038599

[3] Kuo SH , Huang CS, Kuo WH, Cheng AL, Chang KJ, Chia-Hsien CJ. Comprehensive locoregional treatment and systemic therapy for postmastectomy isolated locoregional recurrence. Int J Radiat Oncol Biol Phys 2008;72:1456–64. 18692329 10.1016/j.ijrobp.2008.03.042

[4] McCarthy CM , Pusic AL, Sclafani L, Buchanan C, Fey JV, Disa JJ, et al. Breast cancer recurrence following prosthetic, postmastectomy reconstruction: incidence, detection, and treatment. Plast Reconstr Surg 2008;121:381–8. 18300953 10.1097/01.prs.0000298316.74743.dd

[5] Handel N , Lewinsky B, Jensen JA, Silverstein MJ. Breast conservation therapy after augmentation mammaplasty: is it appropriate? Plast Reconstr Surg 1996;98:1216–24. 8942907 10.1097/00006534-199612000-00015

[6] McIntosh SA , Horgan K. Breast cancer following augmentation mammoplasty – a review of its impact on prognosis and management. J Plast Reconstr Aesthet Surg 2007;60:1127–35. 17613294 10.1016/j.bjps.2007.03.017

[7] Prabhakaran S , Elston JB, Lleshi A, Kumar A, Sun W, Khakpour N, et al. Single institution review of patients with prior breast augmentation undergoing breast conservation therapy for breast cancer. Ann Plast Surg 2017;78:S289-s91–S291. 28328631 10.1097/SAP.0000000000001040

[8] Schwartz J-CD . Use of a bioabsorbable implant-acellular dermal matrix construct to facilitate Oncoplastic breast-conserving surgery. Plast Reconstr Surg Glob Open 2021;9:e3356. 33564586 10.1097/GOX.0000000000003356 PMC7858196

[9] Margulies IG , Salzberg CA. The use of acellular dermal matrix in breast reconstruction: evolution of techniques over 2 decades. Gland Surg 2019;8:3–10. 30842922 10.21037/gs.2018.10.05 PMC6378261

[10] Gigli S , Amabile MI, Di Pastena F, Manganaro L, David E, Monti M, et al. Magnetic resonance imaging after breast oncoplastic surgery: an update. Breast Care 2017;12:260–5. 29070991 10.1159/000477896 PMC5649274

[11] Dialani V , Lai KC, Slanetz PJ. MR imaging of the reconstructed breast: what the radiologist needs to know. Insights Imaging 2012;3:201–13. 22696083 10.1007/s13244-012-0150-7 PMC3369124

[12] Tomouk T , Mahler-Araujo B, Gaskarth MT, Malata CM, Forouhi P. Mastopexy sutures masquerading as an organic breast lesion on MRI scan. J Plast Reconstr Aesthet Surg 2014;67:e182–3. 24559730 10.1016/j.bjps.2014.01.034

[13] Margolis NE , Morley C, Lotfi P, Shaylor SD, Palestrant S, Moy L, et al. Update on imaging of the postsurgical breast. Radiographics 2014;34:642–60. 24819786 10.1148/rg.343135059

